# Gas5 is an essential lncRNA regulator for self-renewal and pluripotency of mouse embryonic stem cells and induced pluripotent stem cells

**DOI:** 10.1186/s13287-018-0813-5

**Published:** 2018-03-21

**Authors:** Jiajie Tu, Geng Tian, Hoi-Hung Cheung, Wei Wei, Tin-lap Lee

**Affiliations:** 1 Institute of Clinical Pharmacology, Key Laboratory of Anti-inflammatory and Immune Medicine, Ministry of Education, Anhui Collaborative Innovation Center of Anti-inflammatory and Immune Medicine, Anhui Medical University, 81# Meishan Road, Hefei, Anhui China; 2 Faculty of Medicine, School of Biomedical Sciences, The Chinese University of Hong Kong, Lo Kwee-Seong Integrated Biomedical Sciences Building, Shatin, N.T 622A Hong Kong, Special Administrative Region of China

**Keywords:** lncRNA, Gas5, mESCs, iPSCs, Self-renewal, Pluripotency

## Abstract

**Background:**

The regulatory role of long noncoding RNAs (lncRNAs) have been partially proved in embryonic stem cells (ESCs) and induced pluripotent stem cells (iPSCs).

**Methods:**

In the current study, we investigated mouse ESC (mESC) self-renewal, differentiation, and proliferation in vitro by knocking down a lncRNA, growth arrest specific 5 (Gas5). A series of related indicators were examined by cell counting kit-8 (CCK-8) assay, quantitative reverse-transcription polymerase chain reaction (qRT-PCR), Western blot, alkaline phosphatase staining, propidium iodide (PI) staining, Annexin V staining, competition growth assay, immunofluorescence, and chromatin immunoprecipitation (ChIP)-qPCR. An in vivo teratoma formation assay was also performed to validate the in vitro results. qRT-PCR, fluorescence-activated cell sorting (FACS), alkaline phosphatase staining, and immunofluorescence were used to evaluate the role of Gas5 during mouse iPSC reprogramming. The regulatory axis of Dicer-miR291a–cMyc-Gas5 and the relationship between Gas5 and Tet/5hmC in mESCs was examined by qRT-PCR, Dot blot, and Western blot.

**Results:**

We identified that Gas5 was required for self-renewal and pluripotency of mESCs and iPSCs. Gas5 formed a positive feedback network with a group of key pluripotent modulators (Sox2, Oct4, Nanog, Tcl1, Esrrb, and Tet1) in mESCs. Knockdown of Gas5 promoted endodermal differentiation of mESCs and impaired the efficiency of iPSC reprogramming. In addition, Gas5 was regulated by the Dicer-miR291a–cMyc axis and was involved in the DNA demethylation process in mESCs.

**Conclusions:**

Taken together, our results suggest that the lncRNA Gas5 plays an important role in modulating self-renewal and pluripotency of mESCs as well as iPSC reprogramming.

**Electronic supplementary material:**

The online version of this article (10.1186/s13287-018-0813-5) contains supplementary material, which is available to authorized users.

## Background

The mammalian genome encodes a vast number of long noncoding RNAs (lncRNAs) which are a class of RNAs increasingly recognized as major players in gene regulation [1]. Like coding mRNAs, most lncRNAs are transcribed by RNA polymerase II, 5′ capped, spliced, and some polyadenylated, but they lack protein-coding potential. Recent studies indicate that some lncRNAs have versatile biological functions under different conditions [2, 3].

As expected, lncRNAs are emerging regulators in embryonic stem cells (ESCs) [4]. Several regulatory lncRNAs for pluripotency have been identified in ESCs based on their specific expression pattern [5]. From recent studies, lncRNAs also appear as regulators for ESC lineage differentiation [6]. Specifically, a lncRNA, Malat1, has been shown to regulate synaptogenesis [7]. Another lncRNA, Braveheart, is required for cardiovascular lineage commitment from mesoderm [8]. The role of lncRNAs in endodermal differentiation from pluripotent ESCs or induced pluripotent stem cells (iPSCs) remains unknown.

Growth arrest specific 5 (Gas5) belongs to the 5’ terminal oligopyrimidine class and is a small nucleolar RNA (C/D box snoRNA genes) host gene [9]. Multiple functions have been associated with this lncRNA, mainly including cell growth and apoptosis [10]. Gas5 is also identified as an essential regulator in cancer [11]. Recently, the role of Gas5 in human ESCs had been reported [12]. However, the role of Gas5 in other pluripotent stem cells (such as mouse ESCs (mESCs) and iPSCs) is still unknown. Here, we reveal an essential role of Gas5 in mESCs and iPSCs. Gas5 is highly conserved in vertebrates. Depletion of Gas5 RNA in mESCs affected a number of genes involved in self-renewal and endodermal differentiation possibly through interaction with the pluripotent transcriptional factors and the DNA demethylation regulator. In addition, proliferation was repressed in Gas5-knockdown mESCs. Consistent with the effect on pluripotency, disruption of Gas5 expression impaired the efficiency of somatic reprogramming to iPSCs. Taken together, our results suggest that Gas5 is required for the maintenance of mESC self-renewal and proliferation by inhibiting endodermal lineage differentiation.

## Methods

### Cell culture

Mouse E14Tg2A ESCs were maintained on 0.1% gelatin-coated culture plates in Dulbecco's modified Eagle's medium (DMEM; GIBCO, New York, USA) supplemented with 15% ES-qualified fetal bovine serum (ES-FBS; GIBCO), 55 mM β-mercaptoethanol (GIBCO), 2 mM l-glutamax (GIBCO), 0.1 mM nonessential amino acid (NEAA; GIBCO), gentamycin (GIBCO), and 1000 U/ml leukemia inhibitory factor (LIF; ESGRO, Millipore, Billerica, USA) under feeder-free condition. Cells were passaged every 2–3 days by dissociation with recombinant trypsin (Sigma, Darmstadt, Germany).

### Published data analysis

Related sequencing data (GSE8024, GSE36114, and GSE26833) were retrieved from the NCBI GEO database.

### Plasmids and miRNA mimics/inhibitors

Gas5, Dicer, and cMyc short hairpin (sh)RNAs were designed using the on-line design program from MIT (http://sirna.wi.mit.edu/home.php). The 19-nucleotide hairpin-type shRNAs with a 9-nucleotide loop were cloned into pSUPER-puromycin (OligoEngine, Seattle, USA) and pLVTHM (Addgene, Cambridge, USA) according to the manufacturers’ protocols. Puromycin selection (pSUPER vector, for reprogramming part) and green fluorescent protein (GFP) sorting (pLVTHM vector) were used to isolate successfully transfected cells. Quantitative reverse-transcription polymerase chain reaction (qRT-PCR) was used for validation of RNA expression. MiR-291a mimics and inhibitors was purchased from GenePharma (Shanghai, China).

### ESC differentiation

For LIF withdrawal and differentiation assays, cells were cultured by the hanging drop method in a 10-cm culture dish and LIF was removed the day after (day 0). After 2 days of embryoid body (EB) formation by the hanging drop method, EBs were transferred to a petri plate for suspension culture up to 8 days. The morphology and number of EBs was also noted. Retinoic acid (RA) or Activin A (Act A) was used to induce specific germ layer differentiation. Ectoderm was induced by RA (10^−9^ M, Sigma) and mesendoderm was induced by different concentrations of Act A (2.5 ng/ml for mesoderm and 50 ng/ml for endoderm inductions; R&D Systems, Minneapolis, USA).

### Immunofluorescence

1 × 10^5^ cells were cultured on a cover glass in a 12-well plate with 700 μl of medium. The cells were allowed to grow to the desired morphology and density before the staining procedure. To stain the cells, cells were first washed once with phosphate-buffered saline (PBS) and fixed by 4% paraformaldehyde/4% sucrose in PBS at room temperature, followed by permeabilization and DNA denaturation by 0.2% TritonX-100 in 4 M HCl. After that, the cells were washed with PBS and blocked in 80 μL bovine serum albumin (BSA; 3%). The cells were incubated by Sox2 (SC-17320, 1:100, Santa Cruz, Dallas, USA) and Gata4 (SC-25310, 1:50, Santa Cruz) in BSA (3%) at 4 °C overnight, and then conjugated with RED-X-conjugated mouse anti-rabbit monoclonal antibody (1:500, Santa Cruz) and 4’,6-diamidino-2-phenylindole (DAPI; 1:1000, Santa Cruz). The glass slides were mounted with a cover slip before imaging.

### Alkaline phosphatase (ALP) staining

ALP activity detection was carried out using the blue-color and red-color AP staining Kits (SBI, Palo Alto, USA) according to the manufacturer’s protocol.

### RNA extraction, cDNA synthesis, and real-time PCR

Total RNA was extracted by Trizol reagent (Invitrogen, Carlsbad, USA) according to a standard protocol. Concentration and quality of all RNA samples were evaluated by Nanodrop 2000 (Thermo, Waltham, USA), and the 260/280 and 260/230 values of all samples were more than 1.8 and 1.9, respectively. Reverse transcription was performed with the MasterMix kit (Takara, Shiga, Japan) following the standard protocol. Quantitative PCR was performed using the Universal SYBR Green Master mix (Applied Biosystems, Waltham, USA) on a StepOnePlus real-time PCR system (Applied Biosystems). Gene expression was normalized to GAPDH unless otherwise stated.

### Western blot

Cells were lysed in SDS buffer. The protein concentration was measured by BCA assay kit (Thermo). Equal amounts of cell lysates were loaded, blotted onto a polyvinylidene difluoride (PVDF) membrane, and probed with the following primary antibodies: Oct4 (SC-8628, 1:1000, Santa Cruz), Sox2 (SC-17320, 1:1000, Santa Cruz), Tet1 (ab191698, 1:500, Abcam, Cambridge, UK), Tet2 (ABE364, 1: 1000, Millipore), and GAPDH (ab8245, 1:4000, Abcam). Glyceraldehyde 3-phosphate dehydrogenase (GAPDH) was used as the loading control. After incubation with the appropriate secondary antibodies, signals were visualized by enhanced chemiluminescence (GE systems, Fairfield, USA).

### ChIP-qPCR

Chromatin immunoprecipitation (ChIP) assays were performed in accordance with the manufacturer’s instructions for the Imprint Chromatin Immunoprecipitation Kit (Sigma). qPCR was consequently performed according to a standard protocol.

### Teratoma formation

mESCs were trypsinized and resuspended at a concentration of 1 × 10^6^ cells/100 μL and injected into nude mice subcutaneously. After approximately 5–8 weeks, teratomas were harvested for qRT-PCR and histologic analysis when tumors exceeded 2.0 cm in diameter and were fixed overnight in 4% paraformaldehyde. Paraffin sections and hematoxylin and eosin (H&E) staining were performed according to a general protocol. Animal handling and maintenance were performed in accordance with institutional guidelines.

### CCK-8 assay

The cell proliferation rate was measured using the cell counting kit-8 (CCK-8; DOJINDO, Tabaru, Japan) according to the manufacturer’s protocol.

### Cell cycle analysis

Cell cycle regulation was determined by a propidium iodide (PI; Sigma) staining assay according to a standard protocol.

### Apoptosis analysis

Apoptosis analysis was performed by a Annexin V/PI (Invitrogen) staining assay according to a standard protocol.

### Competition growth assay

GFP^+^ mESCs (pLVTHM-Gas5 and pLVTHM) and GFP^–^ mESCs (wild-type (WT)) were mixed at a nearly 1:1 ratio and cultured together for two passages. The ratio of GFP^+^ and GFP^–^ cells was determined before and after passaging by a flow cytometer.

### Dot blot

Genomic DNA samples were prepared with twofold serial dilutions in Tris-EDTA (TE) buffer and then denatured in 0.4 M NaOH at 72 °C for 10 mins. Denatured DNA samples were spotted on a PVDF membrane. The membrane was baked at 80 °C for 10 mins and crosslinked by ultraviolet (UV) light for 10 mins. The membrane was then blocked with 5% blocking buffer for 1 h, incubated with 5-hmC primary antibody (39,769, 1:5000, Active Motif, Carlsbad, USA) for 1 h and, after incubation with the horseradish peroxidase (HRP)-conjugated rabbit secondary antibodies (1:10,000, GE systems), signals were visualized by enhanced chemiluminescence.

### iPSC reprogramming

Mouse embryonic fibroblasts (MEFs) were isolated from E13.5 Oct4-GFP mouse embryos and washed in PBS. Reprogramming were performed based on the Lentiviral mediated tet-inducible reprogramming system.

### Statistical analyses

The error bars represent the standard error of mean (SEM) of three independent experiments, and statistically significant differences by Student’s *t* test are indicated by *, **, and ***, indicating *P* < 0.05, *P* < 0.01, and *P* < 0.001, respectively.

## Results

### Characterization of lncRNA Gas5 in ESCs

ESCs are an excellent in vitro model for studying the role of lncRNAs in pluripotent cells and cellular differentiation [13, 14]. To identify lncRNAs participating in mESC pluripotency and lineage differentiation, we analyzed the transcriptome of mESCs during differentiation [15]. Among the differentially expressed lncRNAs, we identified a novel lncRNA, Gas5, that was highly enriched in pluripotent ESCs. The role of this lncRNA in mESCs remains unknown.

**Fig. 1:**
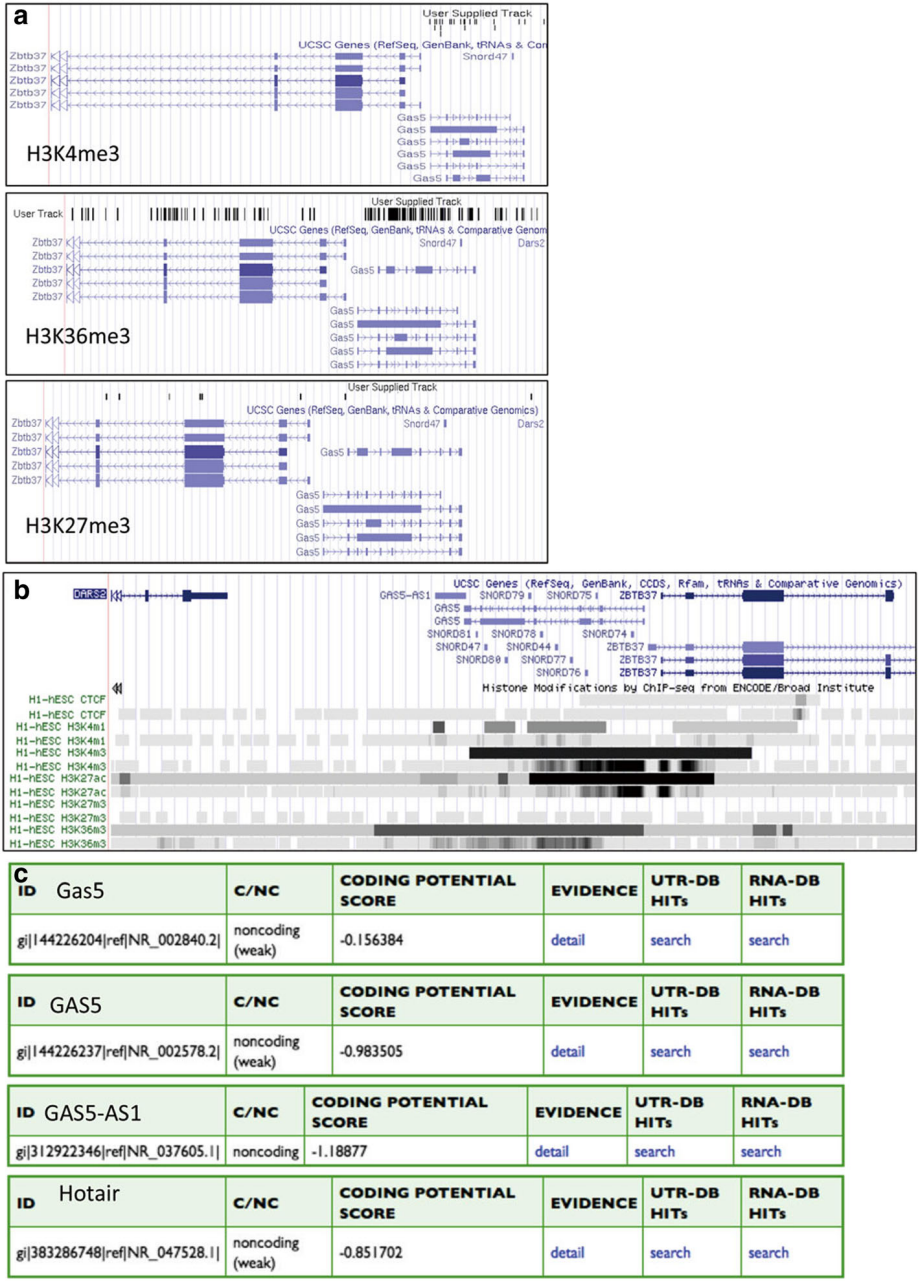
Characterization of Gas5 in ESCs. **a,b** Genome browser plot of histone methylations related to transcriptional activation (H3K4me3 and H3K36me3) and repression (H3K27me3) in the gene body region of Gas5 in both mouse (mESCs) and human (hESCs) embryonic stem cells. **c** The prediction of the protein-coding potential of mouse and human Gas5/GAS5 by Coding Potential Calculator (CPC)

There are at least six isoforms of murine Gas5 (Fig. 1a), including a ~ 2.5-kb major transcript. Interestingly, the promoter sequence of Gas5 is more conserved than that of the gene body across different species (Additional file 1: Figure S1). The genomic locus is enriched with epigenetic marks related to transcriptional activation (H3K4me3 and H3K36me3). Epigenetic marks for active transcription (H3K4me3 and H3K36me3) were found in the gene body region of Gas5, whereas nearly no repressive epigenetic mark (H3K27me3) was found in either mouse or human ESCs (Fig. 1a, b). Thus, Gas5 transcription appears to be tightly regulated by epigenetic mechanisms in ESCs. To confirm that mouse Gas5 is a bona fide noncoding transcript, Coding Potential Calculator (CPC) software was used to calculate the potential protein-coding capacity. The results from CPC analysis suggested that Gas5 has a very low coding potential (Fig. 1c).

### Gas5 is required for mESC self-renewal

**Fig. 2:**
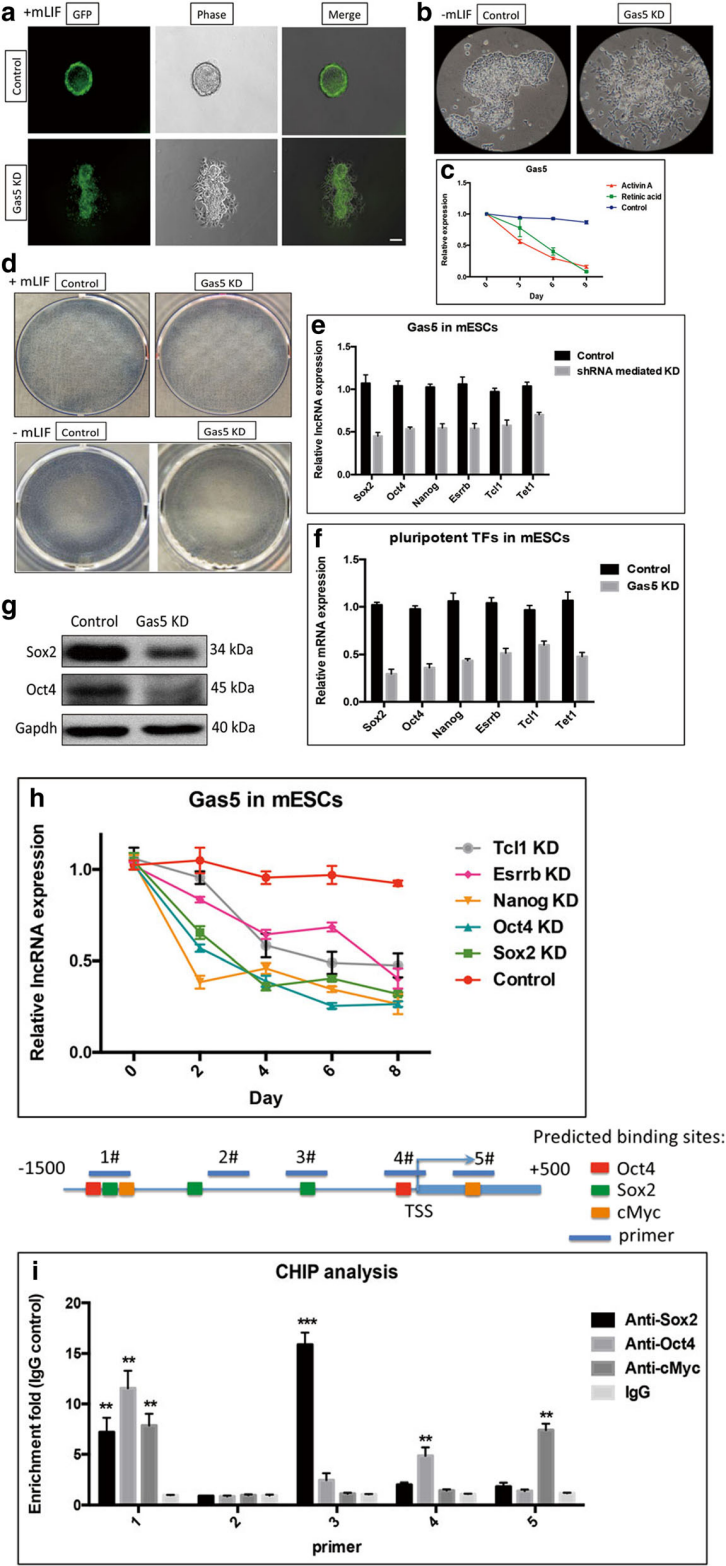
Gas5 is required for mESC self-renewal. **a**, **b** Differentiating morphological change was observed in Gas5 knockdown (KD) mouse embryonic stem cells (mESCs). **c** The endogenous expression of Gas5 expression in Activin A- or retinoic acid-induced differentiation of mESCs. **d** The pluripotency marker ALP staining in Gas5 KD mESCs. **e** Gas5 expression in six important pluripotent transcriptional factors Nanog, Sox2, Oct4, Tcl1, Esrrb, and Tet1 KD mESCs. **f** mRNA expression of six pluripotent transcriptional factors (TFs) Nanog, Sox2, Oct4, Tcl1, Esrrb, and Tet1 in Gas5 KD mESCs. **g** Protein expression of Sox2 and Oct4 in Gas5 KD mESCs. **h** Gas5 expression during a time-elapsed knockdown assay over a period of 8 days following either Sox2, Oct4, Nanog, Esrrb, and Tcl1 KD in mESCs. **i** Chromatin immunoprecipitation (ChIP) analysis of the binding of Oct4, Sox2, and cMyc to Gas5 promoter by using Oct4, Sox2, and cMyc antibodies. *P < 0.05, **P < 0.01, ***P < 0.001, by *t* test, *n* = 3. Error bars represent SEM of the indicated experiment replicates. GFP green fluorescent protein, lncRNA long noncoding RNA, mLIF mouse leukemia inhibitory factor, shRNA short hairpin RNA

We postulated that Gas5 might play a role in maintaining mESC pluripotency and self-renewal, in a similar way to what we know about pluripotency drivers, such as Sox2, Oct4, and Nanog, which are highly expressed in undifferentiated mESCs. We therefore sought to determine if Gas5 is required for ESC pluripotency. To test this, a lentivirus-mediated Gas5 knockdown (KD) was performed in mESCs (Additional file 2: Figure S2). Upon Gas5 KD, mESCs showed spontaneous differentiating phenotype (Fig. 2a, b). The endogenous expression of Gas5 gradually decreased during Act A- or RA-induced differentiation in mESCs (Fig. 2c). ALP, a marker of ESC pluripotency, was significantly repressed in Gas5 KD mESCs, further confirming that Gas5 is required for pluripotency of mESCs (Fig. 2d). Next, to investigate whether Gas5 is regulated by transcription factors known to regulate pluripotency, we detected endogenous expression of Gas5 in mESCs following Sox2, Oct4 Nanog, Esrrb, or Tcl1 knockdown. We chose these five transcriptional factors as they are known to maintain pluripotency in mESCs. According to our qPCR and Western blot results, Gas5 was repressed upon knockdown of all five pluripotent markers (Fig. 2e). We observed the same result by a time-elapsed knockdown assay over a period of 8 days following individual Sox2, Oct4 Nanog, Esrrb, and Tcl1 knockdown (Fig. 2h). Interestingly, expressions of these five transcriptional factors were correspondingly decreased in Gas5 KD mESCs (Fig. 2f and g), suggesting that there may exist a co-regulatory network or a feedback loop between Gas5 and these pluripotent factors in mESCs. By analyzing the JASPAR database, several potential binding sites of Sox2, Oct4, and cMyc were found in Gas5 promoter. Therefore, we performed ChIP to examine the binding of Oct4, Sox2, and cMyc to the Gas5 promoter (Fig. 2i and Additional file 3: Figure S3). The ChIP-qPCR results confirmed the bindings of Sox2, Oct4, and cMyc to the promoter region of Gas5, suggesting that Gas5 transcription is directly modulated by at least these three pluripotent factors.

### Gas5 represses mESC endodermal differentiation

**Fig. 3:**
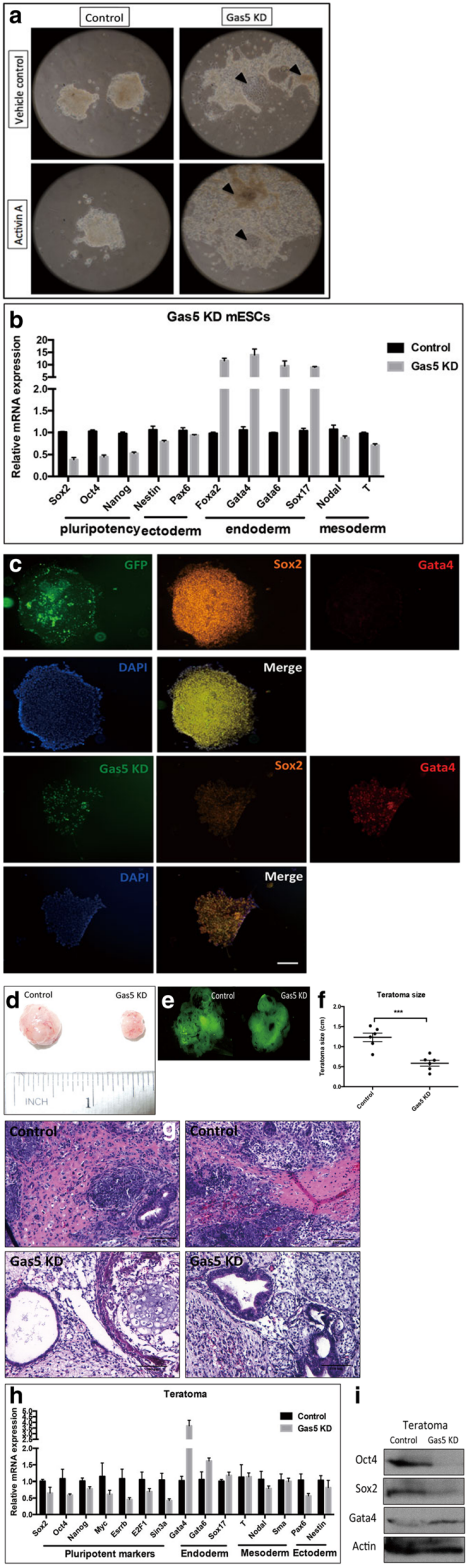
Gas5 represses mESC endodermal differentiation. **a** Typical differentiating phenotype was observed in Gas5 knockdown (KD) mouse embryonic stem cells (mESCs). The arrows in Fig. 3a indicate the typical morphology of differentiated mESCs. **b** Expression of pluripotency and three lineages markers (ectoderm, mesoderm, and endoderm) in Gas5 KD mESCs. **c** Immunofluorescence results of Gata4 (endodermal marker) and Sox2 (pluripotency marker) in Gas5 KD mESCs. **d**–**f** Teratoma formation using Gas5 KD and control mESCs, and the size of Gas5 KD and control ESC-formed teratomas was calculated. **g** Histological analysis (H&E staining) of control and Gas5 KD mESC-formed teratomas. **h** Expression of pluripotency and three lineages markers (ectoderm, mesoderm, and endoderm) in Gas5 KD and control mESC-formed teratomas. **i** Western blot results of Gata4 (endodermal marker), Oct4, and Sox2 (pluripotency marker) in Gas5 KD and control mESC-formed teratomas. ***P < 0.001, *t* test, *n* = 3. Error bars represent SEM of the indicated experiment replicates. DAPI 4',6-diamidino-2-phenylindole, GFP green fluorescent protein

To further evaluate the role of Gas5 during mESC differentiation, we first examined the effect of Gas5 on mESC differentiation using an in vitro differentiation model. Differentiation was observed as early as day 5 in Gas5 KD mESCs (Fig. 3a). We observed that the mESCs started flattening out and detaching from the edges of the colonies. Subsequently, most colonies lost their typical monotype appearance and formed three-dimensional structures with different cell morphologies. Endodermal markers were strongly induced upon Gas5 knockdown (Fig. 3b). In addition, we observed increased Gata4^+^ cells and decreased Sox2^+^ cells in Gas5 KD ESC colonies (Fig. 3c), suggesting that Gas5 plays a role in repressing endodermal lineage differentiation.

In addition, teratoma formation assay was performed to examine the role of Gas5 in modulating germ layer differentiation of mESCs in vivo. Gas5 KD mESCs and control mESCs were subcutaneously injected into nude mice. After approximately 4–6 weeks, both Gas5 KD mESCs and control mESCs formed teratomas (Fig. 3d–f). Histological analysis showed more ectodermal differentiation in teratomas from control mESCs, while Gas5 KD teratomas contained more differentiated endodermal tissues (Fig. 3g). Consistent with the in vitro results, endodermal markers were elevated in Gas5 KD teratomas (Fig. 3h and i). Together, these in vitro and in vivo results suggest that Gas5 KD promotes endodermal differentiation in mESCs.

### Gas5 is required for mESC proliferation under differentiating conditions

**Fig. 4:**
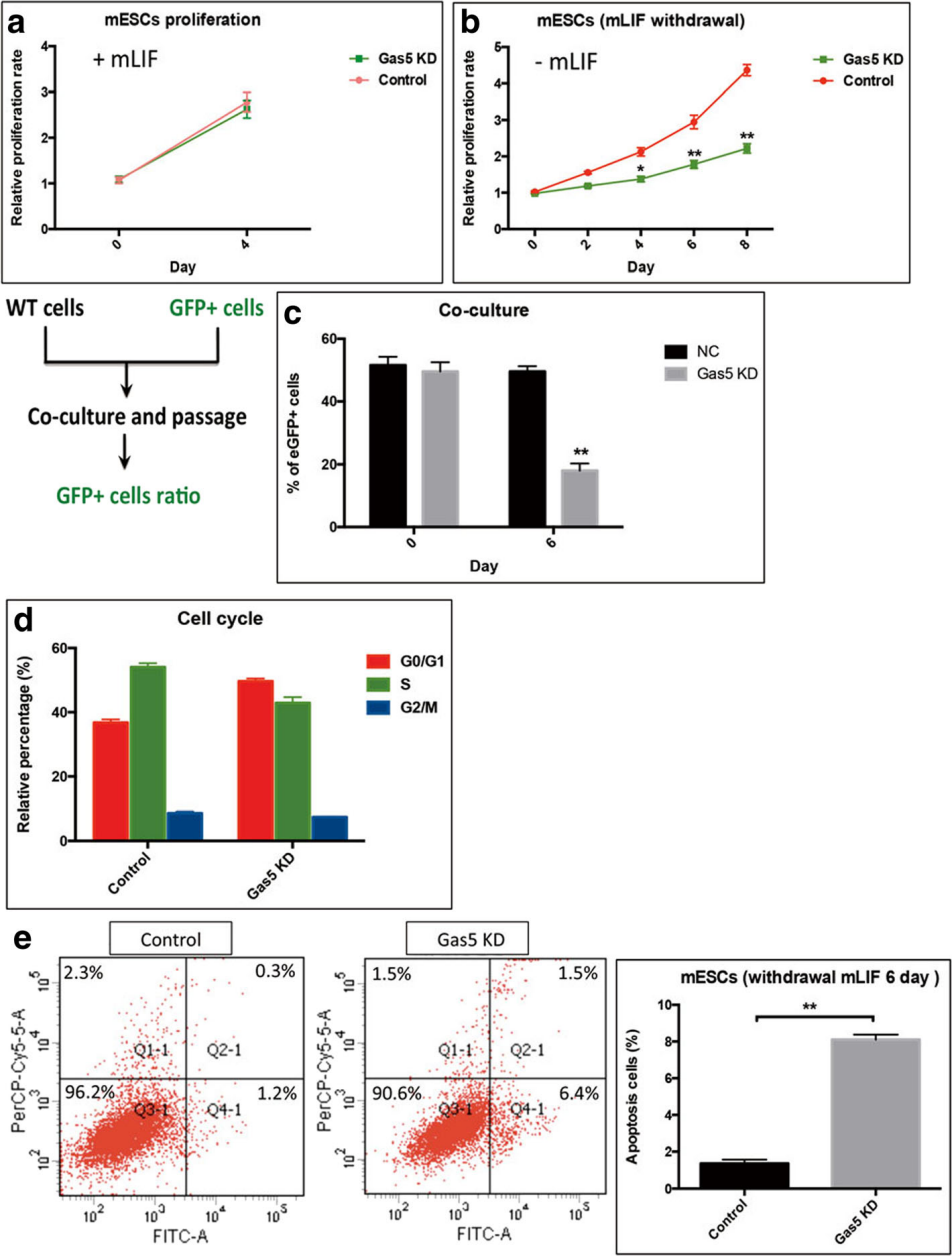
Gas5 maintains the normal proliferation of mESCs under differentiating culture conditions. **a,b** CCK-8 assay results showed the proliferation rate of control and Gas5 knockdown (KD) mouse embryonic stem cells (mESCs) under differentiating culture conditions. **c** Competitive growth assay by mixing mESCs stably expressing control (NC)-green fluorescent protein (GFP) or Gas5 KD-GFP mESCs. **d** PI staining results indicated the cell cycle in Gas5 KD mESCs under differentiating culture conditions. **e** Annexin V/PI staining showed the apoptotic cells in control and Gas5 KD mESCs under differentiating culture conditions. *P < 0.05, **P < 0.01, *t* test, *n* = 3. Error bars represent SEM of the indicated experiment replicates. mLIF mouse leukemia inhibitory factor, WT wild-type

Since Gas5 was originally identified as a cell growth regular, we next assessed the function of Gas5 on mESC proliferation. We observed that the tumor size in Gas5 KD mESC-formed teratomas was much smaller than the control (Fig. 3d), indicating that Gas5 might affect mESCs proliferation. To confirm this, we measured the total cell number of control mESCs and Gas5 KD mESCs by CCK-8 assay in vitro. Under regular growth conditions, Gas5 KD did not affect mESC proliferation (Additional file 4: Figure S4). However, under LIF withdrawal conditions (LIF is the essential factor to maintain mESCs in an undifferentiated status), the Gas5 KD mESCs grew much slower than the controls (Fig. 4b), suggesting that Gas5 is required for mESC growth/proliferation under differentiating culture conditions.

To further verify this observation, we performed a competitive growth assay by mixing mESCs stably expressing control-GFP or Gas5 KD-GFP with the same number of WT mESCs. The percentage of GFP^+^ mESCs before passaging was approximately 50% of the total cells. After three passages, the percentage of mESCs expressing control-GFP remained almost the same, but the percentage of Gas5 KD mESCs was decreased to approximately 20% (Fig. 4c). These data indicated that Gas5 is important for mESC growth under differentiating conditions.

Self-renewing ESCs usually have a long S phase in a cell cycle and a low apoptotic rate. Our data showed that knockdown of Gas5 impairs mESC proliferation. We thus further hypothesized that Gas5 regulates the cell cycle and/or apoptosis in mESCs. The PI staining results indeed showed that Gas5 KD increased the proportion of cells in the G0/G1 phase, with a concomitant decrease in the S and G2/M phases (Fig. 4d). Furthermore, as shown in Fig. 4e, Gas5 KD mESCs exhibited a significantly higher apoptotic rate when compared with control mESCs under LIF withdrawal conditions for 6 days. Together, these data indicate that Gas5 prevents mESCs from apoptosis under differentiating conditions.

### Gas5 is regulated by the Dicer-miR291a–cMyc pathway in ESCs

**Fig. 5:**
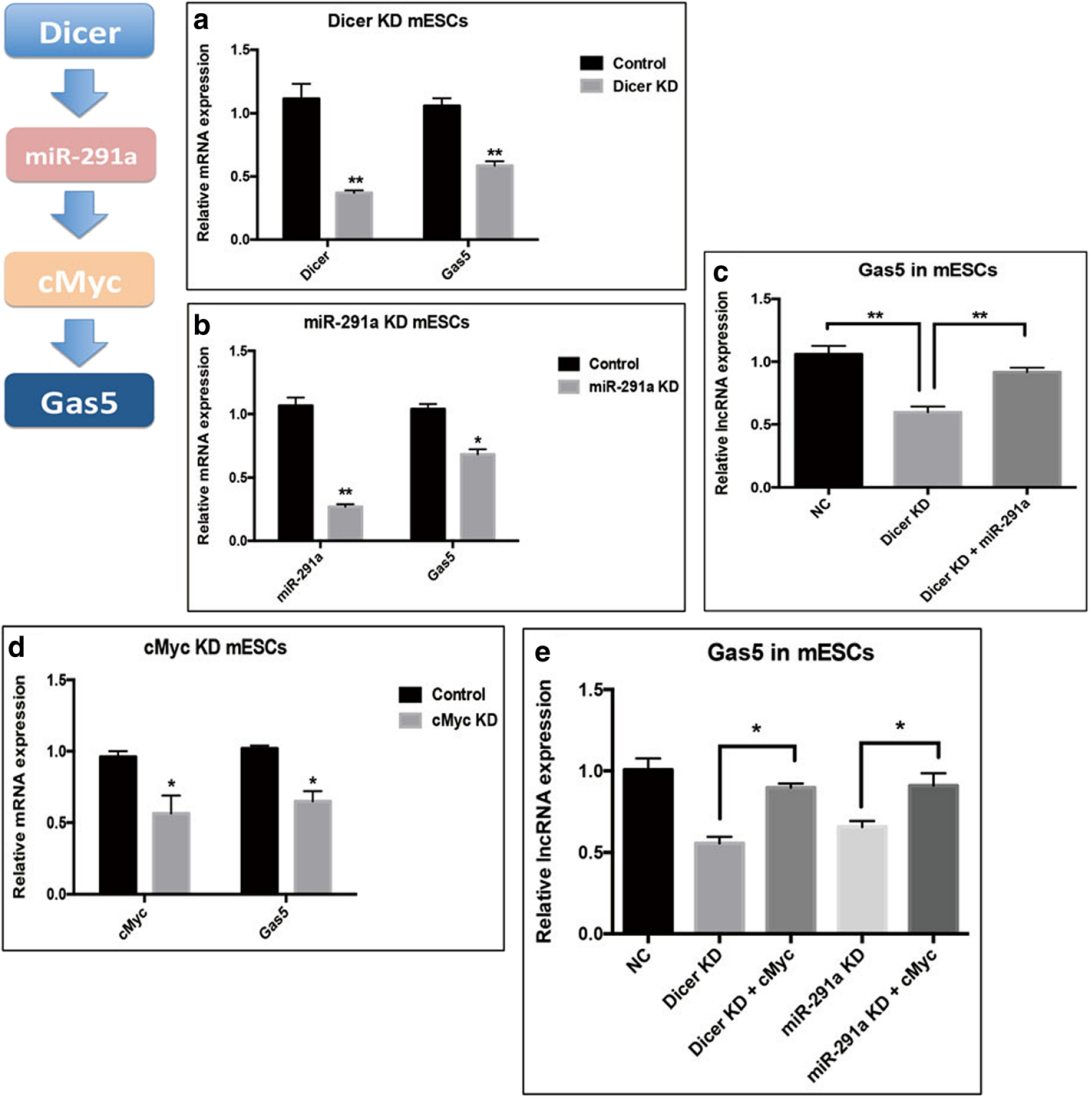
Gas5 is regulated by the Dicer-miR291a–cMyc pathway in ESCs. **a** The expression of Gas5 in Dicer knockdown (KD) mouse embryonic stem cells (mESCs). **b** The expression of Gas5 in miR-291a KD mESCs **c** Overexpression of miR-291a restores the expression of Gas5 in Dicer knockdown mESCs. **d** The expression of Gas5 in cMyc KD mESCs. **e** Enforced expression of cMyc rescues the expression of Gas5 in Dicer and miR-291a KD mESCs. *P < 0.05, **P < 0.01, *t* test, *n* = 3. Error bars represent SEM of the indicated experiment replicates. lncRNA long noncoding RNA, NC normal control

Recent reports have shown that another group of noncoding RNAs, microRNAs (miRNAs), could interact with lncRNAs in mESCs [16, 17]. To determine whether loss of Dicer (an important endonuclease for miRNA biogenesis) affects Gas5 expression, shRNA was used to knockdown Dicer in mESCs. As expected, the expression of Gas5 significantly decreased in Dicer knockdown mESCs (Fig. 5a). We next examined whether the canonical Dicer-miRNA pathway was required for Gas5 in mESCs. Since the miR-290 cluster is the major miRNA in mESCs and it is known that the miR-290 cluster is Dicer-dependent in mESCs [18], we hypothesized that loss of the miR-290 cluster might also affect Gas5. Indeed, Gas5 was repressed in miR-291a (the most abundant miRNA of the miR-290 cluster in mESCs) KD mESCs (Fig. 5b). Furthermore, overexpression of miR-291a in Dicer knockdown mESCs rescued the expression of Gas5 (Fig. 5c). Taken together, these data suggest a possible Dicer-miR291a regulatory pathway for the endogenous Gas5 expression in mESCs.

cMyc is another important transcriptional factor essential for mESC pluripotency, and its expression decreases upon Dicer loss, an effect due to loss of miR-290 cluster expression as documented previously [19]. From our experiments, cMyc KD mESCs showed decreased Gas5 expression, similar to what we observed in Dicer KD and miR-291a KD mESCs (Fig. 5d). Conversely, forced expression of cMyc in Dicer or miR-291a KD mESCs could counteract the downregulation of Gas5 (Fig. 5e). As shown in Fig. 2h, cMyc could directly bind to the promoter of Gas5 and regulate its transcription. Overall, these results suggest that Gas5 is regulated by the Dicer-miR-291a–cMyc axis in mESCs.

### Gas5 promotes iPSC reprogramming efficiency

**Fig. 6:**
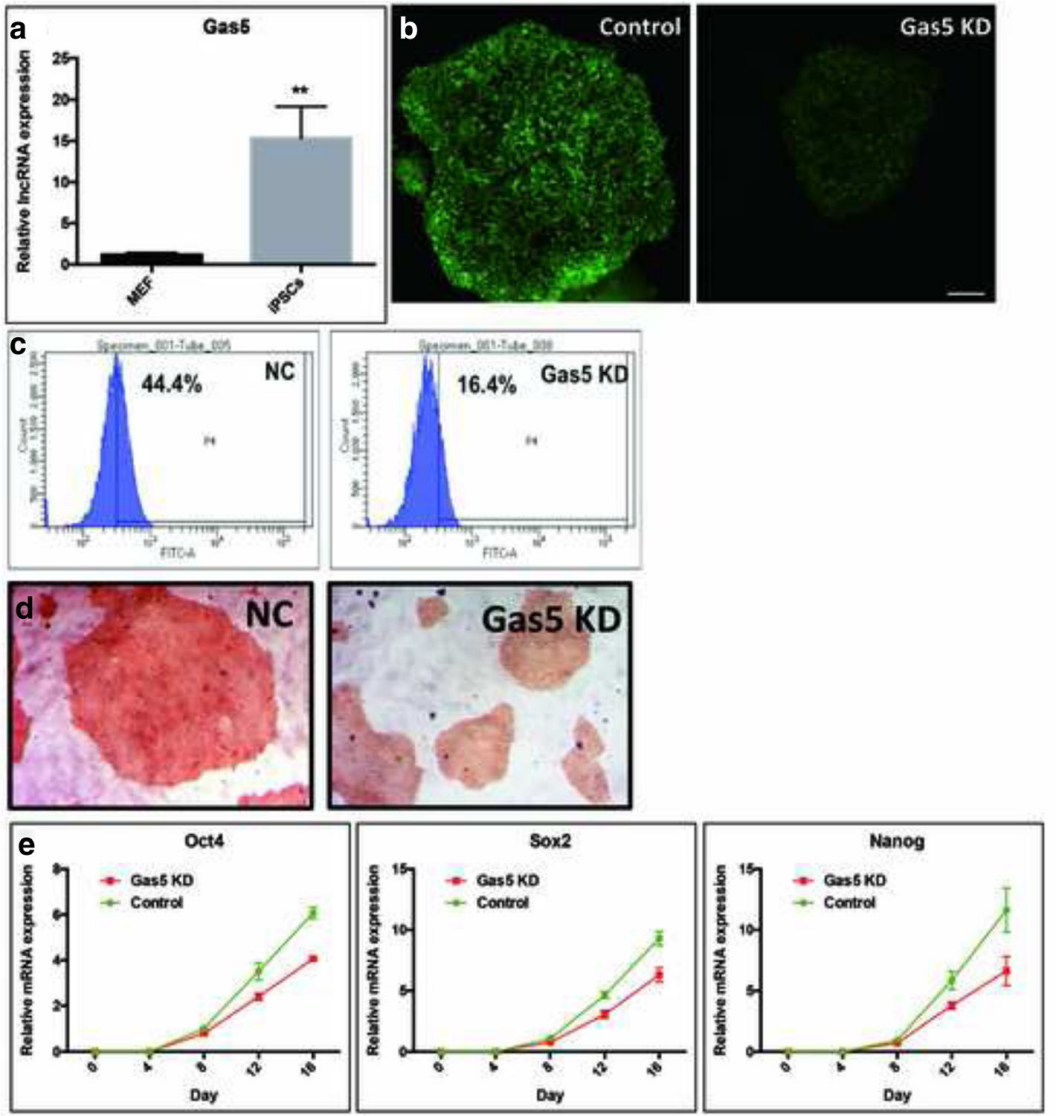
Gas5 induces iPSC reprogramming efficiency. **a** Endogenous expression of Gas5 in mouse embryonic fibroblasts (MEFs) and reprogrammed induced pluripotent stem cells (iPSCs). **b,c** Morphology and quantitative analysis of Oct4-GFP^+^ cells during Gas5 knockdown (KD) and control (NC) MEF reprogramming. **d** Alkaline phosphatase staining results in Gas5 KD-MEF-formed iPSC clones and control MEF-formed iPSC clones. **e** Endogenous expression of pluripotent markers Oct4, Sox2, and Nanog during Gas5 KD MEF- and control MEF-mediated reprogramming. **P < 0.01, *t* test, *n* = 3. Error bars represent SEM of the indicated experiment replicates

We have demonstrated the function of Gas5 on mESC self-renewal and pluripotency. Following our hypothesis, we suggest that this lncRNA might also play a role in reprogramming. We firstly compared endogenous expression of Gas5 in WT MEFs and the corresponding reprogrammed iPSCs. Results showed that Gas5 was significantly increased by approximately 15-fold in iPSCs (Fig. 6a). To investigate whether Gas5 has a beneficial role in reprogramming, we first repressed Gas5 in Oct4-GFP MEFs, followed by ectopic expression of the four Yamanaka factors (Oct4, Sox2, Klf4, and c-Myc). The result shows that Gas5 KD in Oct4-GFP MEFs significantly impaired the reprogramming efficiency, as shown by the number of Oct4:GFP-positive iPSC colonies and ALP-positive cells (Fig. 6b–d). At the same time, diminished expression of key pluripotent factors, such as Oct4, Sox2, and Nanog, was observed in iPSCs reprogrammed from Gas5 knockdown MEFs (Fig. 6e). These data indicate that Gas5 is beneficial for reprogramming to iPSCs.

### Gas5 interacts with the Tet family and regulates 5hmC in ESCs

**Fig. 7:**
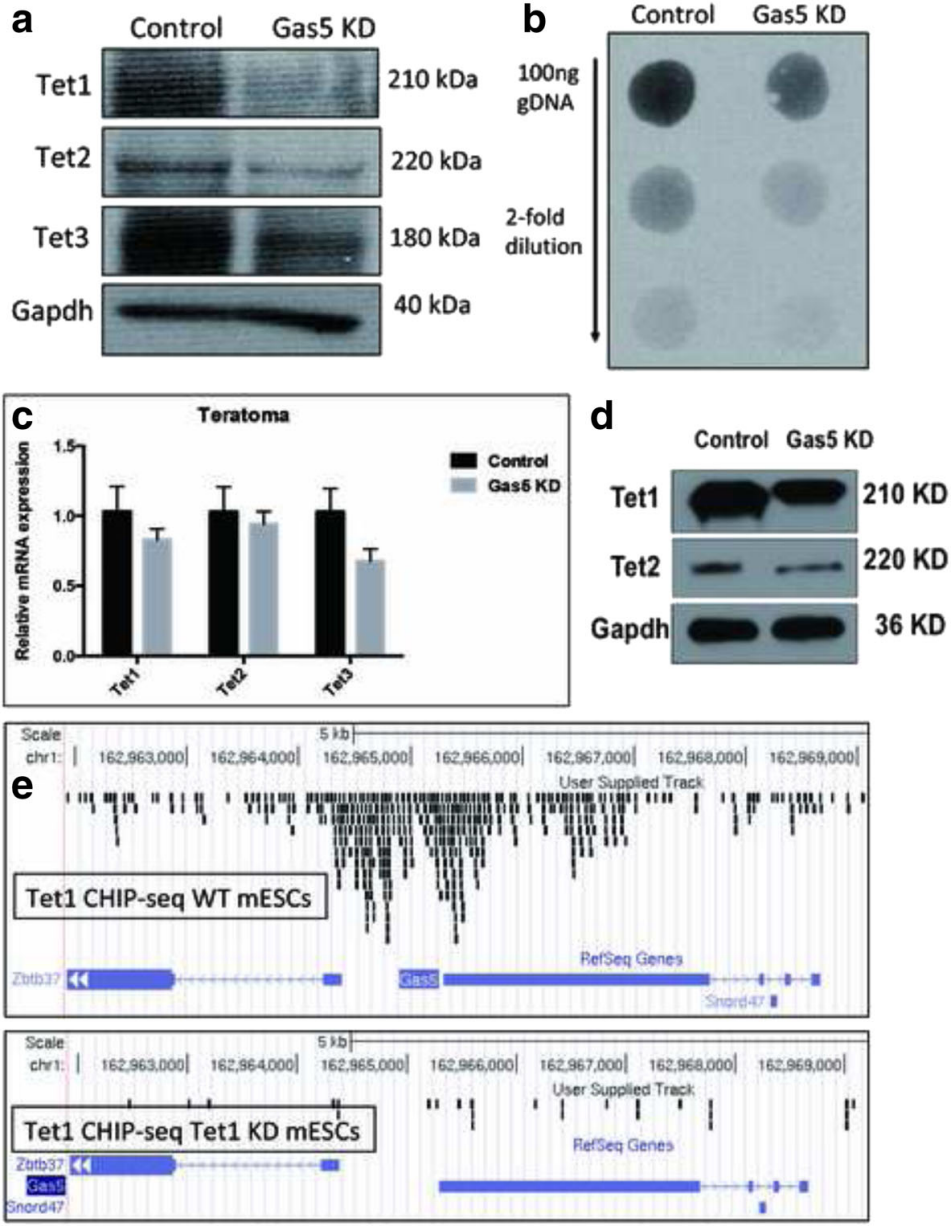
Gas5 interacts with the Tet family and regulates 5hmC in mESCs. **a** Expression of all three Tets (Tet1, Tet2, and Tet3) in Gas5 knockdown (KD) mouse embryonic stem cells (mESCs) at the protein level. **b** The Tet family mediated oxidation production, 5hmC levels in Gas5 KD mESCs. **c**,**d** Expression of all three Tets in Gas5 KD mESC- and control mESC-formed teratomas. **e** The binding signal of Tet1 at the Gas5 promoter region in WT and Tet1 KD mESCs. *P < 0.05, **P < 0.01, ***P < 0.001, *t* test, *n* = 3. Error bars represent SEM of the indicated experiment replicates. CHIP chromatin immunoprecipitation, GAPDH glyceraldehyde 3-phosphate dehydrogenase, WT wild-type

We have found that the expression of methylcytosine dioxygenase Tet1 (Ten-eleven translocation protein 1) was repressed in Gas5 KD mESCs, whereas Gas5 was also downregulated in Tet1 KD mESCs (Fig. 2). Since Tet1 catalyzes the conversion of 5mC (5-methylcytosine) to 5hmC (5-hydroxmethylcytosine) and since Tet1/5hmC is considered as a pluripotent marker in mESCs [20], the mutual regulatory relationship between Gas5 and Tet1 prompts us to suggest an epigenetic role of Gas5 in the pluripotency of mESCs. First, we examined the expression of the three known Tet family members and total 5hmC levels in Gas5 KD mESCs. Western blots showed that all the three Tet proteins (Tet1, Tet2, and Tet3) were decreased in Gas5 KD mESCs (Fig. 7a). Consistently, the total 5hmC level was also decreased (Fig. 7b). Next, we examined the Tet mRNA/protein in teratomas formed in vivo from Gas KD mESCs. All the Tet transcripts and proteins were found decreased in Gas5 KD teratomas (Fig. 7c, d), except that Tet3 could not be detected by Western blot (Fig. 7d). To investigate whether there is a direct binding of Tet to the Gas5 regulatory region, we analyzed the Tet1 ChIP-seq data in mESCs from a previous study [21]. We found that the Tet1 binding signal was enriched at the putative promoter region of Gas5, whereas this signal was significantly reduced in Tet1 KD mESCs (Fig. 7e). These findings together suggest that Gas5 plays an epigenetic role in maintaining pluripotency of mESCs with potential interaction with Tet family/5hmC. Gas5 might also affect mESC self-renewal and differentiation at the epigenetic level. It will be interesting to investigate the other related functions and specific mechanism of this epigenetic regulation.

## Discussion

Embryonic stem cells (ESCs) can self-renew indefinitely in vitro and differentiate into all germ layers [22]. Understanding the molecular mechanisms required for ESCs to maintain a balance between pluripotency and differentiation is critical for advancing stem cell-based therapies in regenerative medicine. It is evident that a number of lncRNAs plays their roles in the mammalian transcriptome [23] via versatile mechanisms [24]. Some lncRNAs regulate networks that contribute to ESC self-renewal and differentiation [25]. For example, it was shown that many lncRNAs are involved in ESC function by two RNAi screens. One study shows that knocking down 26 out of 147 lncRNAs results in decreased expressions of pluripotency markers [6]. Another genome-scale RNAi screen of 1280 lncRNAs in ESCs revealed 20 lncRNAs that are involved in the maintenance of pluripotency [15]. Interestingly, Gas5 was found in the candidate list from both studies, suggesting that Gas5 might be critical for ESC pluripotency. On the other hand, whilst we were preparing this manuscript, another group showed a similar function of Gas5 in human ESCs [12], additionally supporting our findings in the current study. Moreover, we further identified a role of Gas5 in mESC endodermal differentiation, iPSC reprogramming, and epigenetic regulation, all of which have not been reported before.

In this study, we found that KD of Gas5 resulted in loss of pluripotency. Consistent with this, proliferation was decreased in Gas5 KD mESCs. In addition, the cell cycle was altered and apoptosis was induced in Gas5 KD mESCs. These findings suggest that Gas5 may influence the cell cycle regulatory network of mESCs, a possibility consistent with the known involvement of the cell cycle machinery in the establishment and maintenance of the pluripotent state in mESCs [26]. Apart from being indispensable for the self-renewal of mESCs, our data also imply an indispensable role of Gas5 in repression of endodermal differentiation in mESCs.

Although lncRNAs have been previously linked to stem cell pluripotency [6], we report for the first time that the lncRNA Gas5 could affect the “key pluripotent network” in ESCs: Sox2, Oct4, Nanog, Tcl1, Esrrb, and even the epigenetic regulator Tet family. These results suggest the master regulatory role of Gas5 in maintaining pluripotency of mESCs. Further experiments are required to understand the precise mechanism by which Gas5 acts, and identification of proteins that interact with Gas5 in ESCs may help to elucidate its function.

Many epigenetic regulators, including Polycomb group proteins and DNA methyltransferases, are critical for ESC differentiation [27, 28]. Therefore, Gas5-mediated chromatin and transcriptional regulation in lineage differentiation further highlights the importance of chromatin dynamics in cell-fate transitions. From the present study, some lncRNAs might have (biological) functions in ESCs through epigenetic regulations. More studies are required to elucidate the specific mechanisms, such as genome-wide comparison of DNA or histone modifications between specific lncRNA knockout and overexpression in ESCs. The current study first shows that a lncRNA is necessary to repress endoderm differentiation, suggesting that deregulation of Gas5 expression may contribute to endodermal-related disorders.

Many lncRNAs contribute to the epigenetic regulation of gene expression by serving as modular scaffolds for histone modification complexes. We found that Gas5 is regulated by bi-epigenetic regulations (DNA demethylation and histone methylation in ESCs). Gas5 is marked by H3K4me1, H3K4me3, and H3K36me3 modifications in both mouse and human ESCs and there is no repressive mark H3K27me3 deposit at the same genomic locus. The expression of Gas5 gradually decreases during ESC differentiation [29]. We also found that Gas5 mutually promotes Tet family enzymes in mESCs. The diverse roles of these proteins suggest that Gas5 may regulate gene expression through multiple mechanisms in mESCs. Chromatin modification of Gas5 in undifferentiated ESCs appears to demarcate three chromatin domains containing constitutively active or developmentally regulated genes.

Therefore, like some other lncRNAs, Gas5 may contribute to the ‘fine-tuning’ scaffold of chromatin rather than acting as a regulator of gene transcription. Facile and efficient manipulation of genomic segments should help to elucidate the subtle *cis*- and *trans*-regulatory roles of lncRNAs, leading to a better understanding of the evolutionary and functional mechanisms of lncRNAs. However, during establishment of the pluripotent state, it is unclear whether Gas5 transcription might promote an open chromatin conformation to prime future activation of its target genes during differentiation. This question should be our next step to further elucidating the role of Gas5 in mESCs.

## Conclusion

Collectively, we demonstrated the function of Gas5 in mESC self-renewal, endodermal differentiation, and iPSC reprogramming, suggesting another layer of complexity in the networks controlling stem cell biology. Gas5 interacts with key nuclear proteins and epigenetic modifications to exert their biological functions in mESCs.

## Additional files

Additional file 1: **Figure S1.** The promoter region of Gas5 is more conserved than that of the gene body across species. (PDF 1365 kb)

Additional file 2: **Figure S2.** qPCR results show the knockdown efficiency of lentivirus-mediated Gas5 knockdown in mESCs. *P < 0.05, **P < 0.01, ***P < 0.001, *t* test, *n* = 3. Error bars represent SEM of the indicated experiment replicates. (PDF 1365 kb)

Additional file 3: **Figure S3.** Binding site prediction results of Sox2, Oct4, and Nanog in the promoter region of Gas5. (PDF 12625 kb)

Additional file 4: **Figure S4.** Gas5 KD did not affect mESC proliferation under regular culture conditions (with LIF). (PDF 730 kb)
