# Supplementary figures and images for: Gas5 is an essential lncRNA regulator for self-renewal and pluripotency of mouse embryonic stem cells and induced pluripotent stem cells
Source: Stem Cell Res Ther. 2018 Mar 21;9:71. doi: 10.1186/s13287-018-0813-5 (PMC5863440; doi:10.1186/s13287-018-0813-5)

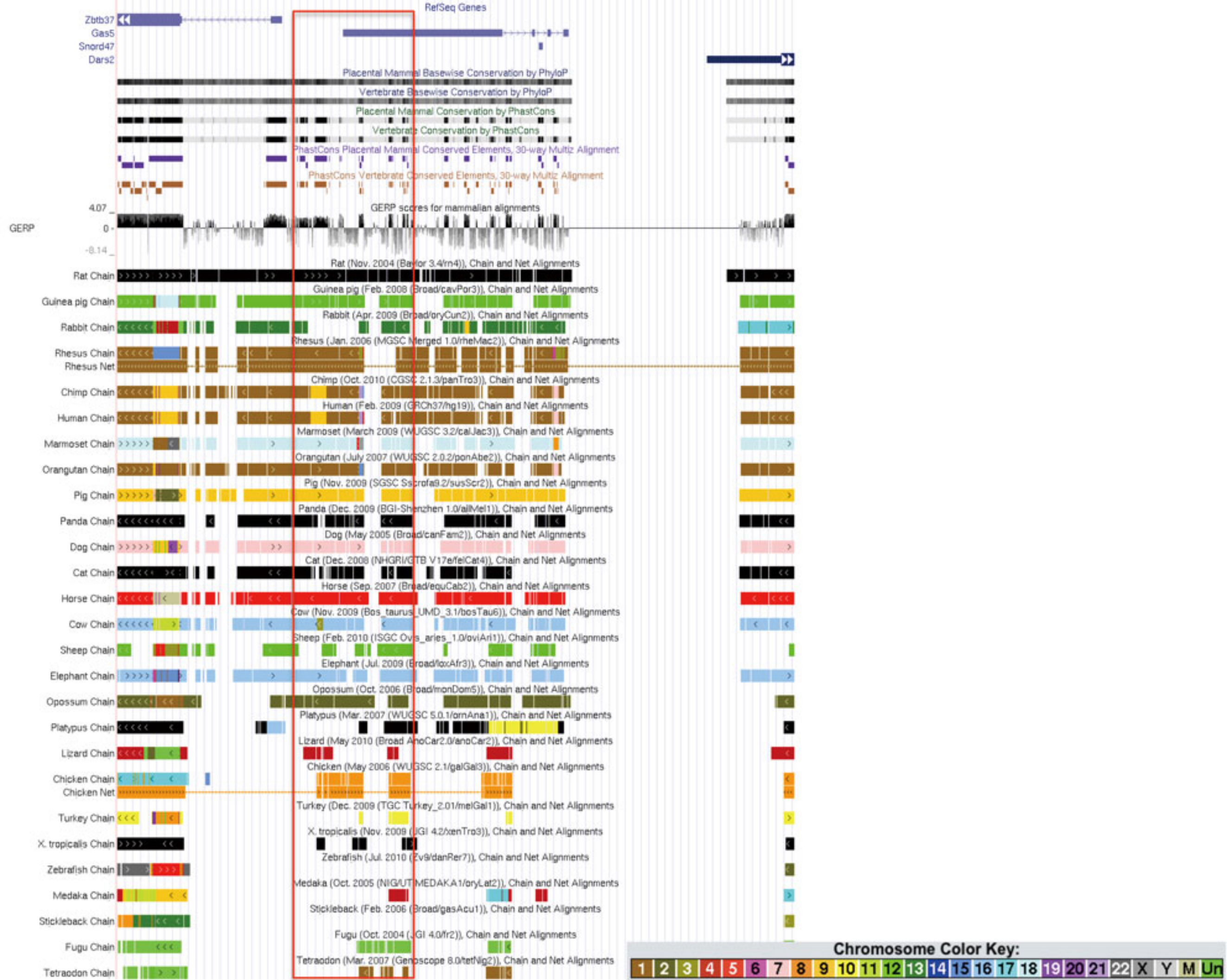

Supplement: Supplementary file 1 — Figure S1. The promoter region of Gas5 is more conserved than that of the gene body across species. (PDF 1365 kb) [file 13287_2018_813_MOESM1_ESM.pdf]

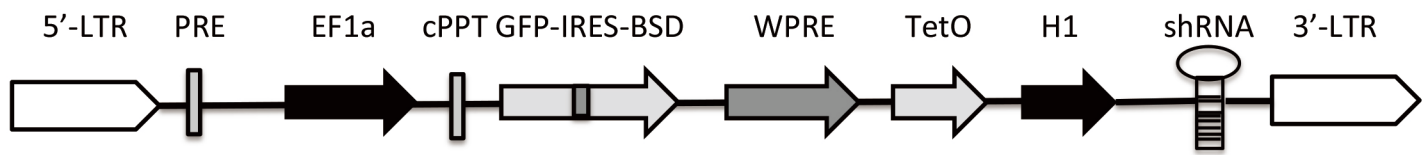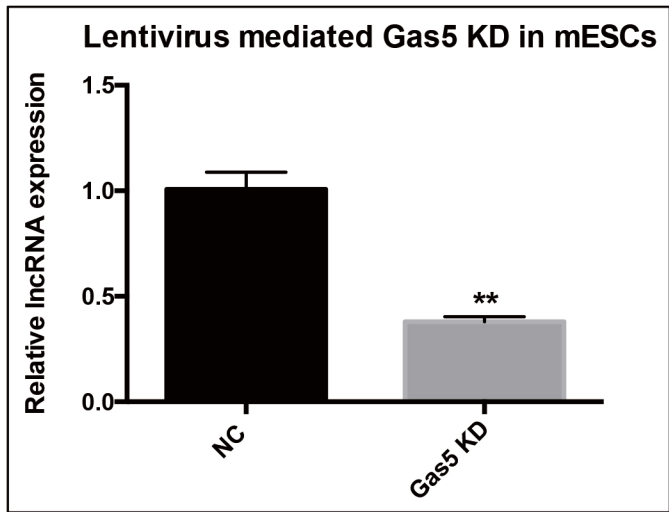

Supplement: Supplementary file 2 — Figure S2. qPCR results show the knockdown efficiency of lentivirus-mediated Gas5 knockdown in mESCs. *P < 0.05, **P < 0.01, ***P < 0.001, t test, n = 3. Error bars represent SEM of the indicated experiment replicates. (PDF 1365 kb) [file 13287_2018_813_MOESM2_ESM.pdf]

# mESCs proliferation

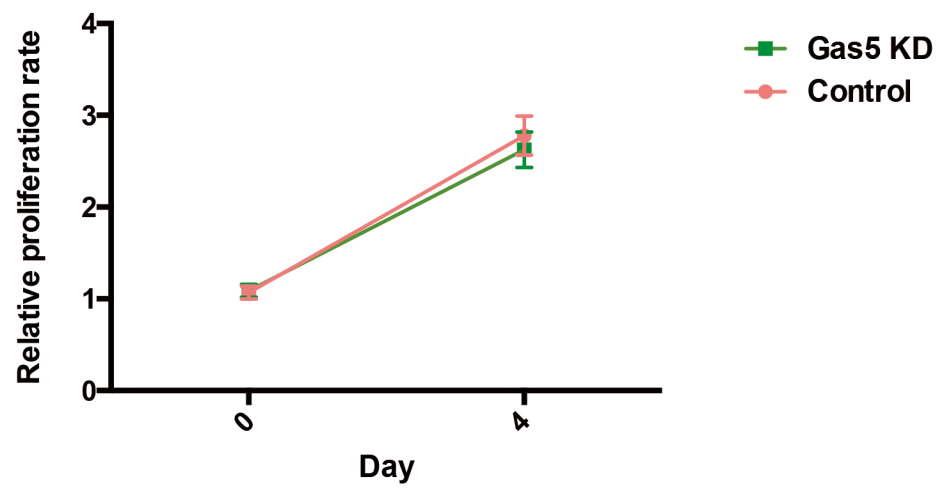

Supplement: Supplementary file 4 — Figure S4. Gas5 KD did not affect mESC proliferation under regular culture conditions (with LIF). (PDF 730 kb) [file 13287_2018_813_MOESM4_ESM.pdf]
