# Supplementary material for: Gas5 is an essential lncRNA regulator for self-renewal and pluripotency of mouse embryonic stem cells and induced pluripotent stem cells
Source: Stem Cell Res Ther. 2018 Mar 21;9:71. doi: 10.1186/s13287-018-0813-5 (PMC5863440; doi:10.1186/s13287-018-0813-5)
Supplement: Supplementary file 3 — Figure S3. Binding site prediction results of Sox2, Oct4, and Nanog in the promoter region of Gas5. (PDF 12625 kb) [file 13287_2018_813_MOESM3_ESM.pdf]

12 putative sites were predicted with these settings (80%) in sequence named **mm10_dna**

Model ID	Model name	Score	Relative score	Start	End	Strand	predicted site sequence
MA0147.2	Myc	2.312	0.808753288823443	226	235	-1	CCTTGTGGTA
MA0147.2	Myc	2.764	0.815387515152996	767	776	-1	ACCTGTGCGC
MA0147.2	Myc	2.417	0.810294425470795	1173	1182	-1	GCACGTCGCT
MA0147.2	Myc	3.708	0.829243067487283	1175	1184	1	CGACGTGCCG
MA0147.2	Myc	2.509	0.811644754723713	1608	1617	-1	GCTTGTGCCC
MA0147.2	Myc	2.128	0.806052630317607	1800	1809	-1	GCACGAGGTG
MA0059.1	MAX::MYC	8.761	0.820018255684475	1801	1811	-1	GTGCACGAGGT
MA0147.2	Myc	4.567	0.841851033011809	1802	1811	1	CCTCGTGCAC
MA0059.1	MAX::MYC	10.612	0.866821922870984	1835	1845	1	AGCCACGCGGT
MA0147.2	Myc	5.230	0.851582210127944	1835	1844	-1	CCGCGTGGCT
MA0059.1	MAX::MYC	8.162	0.804872174298889	1836	1846	-1	CACCGCGTGGC
MA0147.2	Myc	4.940	0.847325737482878	1837	1846	1	CCACGCGGTG

13 putative sites were predicted with these settings (85%) in sequence named **mm10_dna**

Model ID	Model name	Score	Relative score	Start	End	Strand	predicted site sequence
MA0142.1	Pou5f1::Sox2	12.330	0.852552694229211	17	31	1	AATTCACATGCAGAG
MA0147.2	Myc	5.700	0.858480631311329	18	27	-1	GCATGTGAAT
MA0143.3	Sox2	6.426	0.866245447682085	273	280	1	CCAATGTT
MA0143.3	Sox2	5.849	0.856009972708171	422	429	1	CCTTTTTC
MA0143.3	Sox2	5.849	0.856009972708171	473	480	1	CCTCTGTC
MA0143.3	Sox2	6.693	0.870981793987171	527	534	1	CCTTTTTT
MA0143.3	Sox2	5.849	0.856009972708171	953	960	-1	CCTTTGGC
MA0792.1	POU5F1B	9.498	0.893480897244067	1531	1539	1	TATGCAATT
MA0143.3	Sox2	5.849	0.856009972708171	1708	1715	-1	TCTTTGTC
MA0059.1	MAX::MYC	10.612	0.866821922870984	1853	1863	1	AGCCACGCGGT
MA0147.2	Myc	5.230	0.851582210127944	1853	1862	-1	CCGCGTGGCT
MA0143.3	Sox2	5.849	0.856009972708171	1918	1925	1	CCTTAGTC
MA0143.3	Sox2	6.693	0.870981793987171	1929	1936	-1	TCTTTGTT

Comment: This type of analysis has a high sensitivity but abysmal selectivity. In other words: while true functional will be detected in most cases, most predictions will correspond to sites bound in vitro but with no function in vivo. A number of additional contraints of the analysis can improve the prediction; phylogenetic footprinting is the most common. We recommend using the ConSite service, which uses the JASPAR datasets.

The review Nat Rev Genet. 2004 Apr;5(4):276-87 gives a comprehensive overview of transcription binding site prediction
